# Finite-element modelling of elastic wave propagation and scattering within heterogeneous media

**DOI:** 10.1098/rspa.2016.0738

**Published:** 2017-01

**Authors:** A. Van Pamel, G. Sha, S. I. Rokhlin, M. J. S. Lowe

**Affiliations:** 1 Department of Mechanical Engineering, Imperial College London, Exhibition Road, London SW7 2AZ, UK; 2 Department of Materials Science and Engineering, Edison Joining Technology Center, The Ohio State University, 1248 Arthur E. Adams Drive, Columbus, OH 43221, USA

**Keywords:** elastodynamics, wave scattering, stochastic finite elements, heterogeneous media

## Abstract

The scattering treated here arises when elastic waves propagate within a heterogeneous medium defined by random spatial fluctuation of its elastic properties. Whereas classical analytical studies are based on lower-order scattering assumptions, numerical methods conversely present no such limitations by inherently incorporating multiple scattering. Until now, studies have typically been limited to two or one dimension, however, owing to computational constraints. This article seizes recent advances to realize a finite-element formulation that solves the three-dimensional elastodynamic scattering problem. The developed methodology enables the fundamental behaviour of scattering in terms of attenuation and dispersion to be studied. In particular, the example of elastic waves propagating within polycrystalline materials is adopted, using Voronoi tessellations to randomly generate representative models. The numerically observed scattering is compared against entirely independent but well-established analytical scattering theory. The quantitative agreement is found to be excellent across previously unvisited scattering regimes; it is believed that this is the first quantitative validation of its kind which provides significant support towards the existence of the transitional scattering regime and facilitates future deployment of numerical methods for these problems.

## 1. Introduction

Numerical- or grid-based methods [1–3] have found a wide range of applications to solve elastic wave propagation problems arising in seismology, medical ultrasound and non-destructive evaluation. As the availability of computational resource continues to grow, so do the opportunities to deploy these methods to study increasingly complex interactions, such as those encountered by seismic waves scattering within the heterogeneous Earth [4], or by ultrasonic waves scattering from cancellous bone [5] and polycrystalline microstructures of metallic materials [6]. An improved understanding of these phenomena enables a better interpretation of waves, through, for example, imaging algorithms, and hence increases our ability to characterize and detect remote bodies or structural features.

Scattering treated here arises when waves propagate within a heterogeneous medium defined by random spatial fluctuation of its elastic properties. This process is typically characterized by the non-dimensional propagation constant, *ka*, where *k* denotes the wavenumber, and *a* denotes the length scale of the heterogeneity; the various scattering behaviours of the Rayleigh, transitional and stochastic regimes as a function of *ka* are described in [7,8]. In this study, we consider *ka* in the region of 10^−1^ to 10^1^ which embodies all three scattering regimes. These scattering *ka* domains are practically encountered on a millimetre scale for both wavelength and heterogeneity within ultrasonic non-destructive evaluation (NDE), and similarly on seismic length scales approximately five to six orders of magnitude larger [9].

This subject of scattering has received plentiful analytical study [4–8,10–15] among many others and activities remain ongoing [16–20]. Examples of well-established models for propagation within polycrystalline materials are the Stanke & Kino [8] and Weaver-type [14] second-order models (SOMs). The Weaver model, on which the SOM is based, is an extension of the Dyson (for the mean field) and Bethe–Salpeter (mean intensity) [14,21] formalisms found in electromagnetics. Such theoretical models describe propagation and scattering in terms of the perturbed complex wave propagation constant denoting the scattering-induced attenuation, *α*, and velocity, *V*, dispersion characteristics for a spatially coherent plane wave. To arrive at a solution, however, it is often necessary to approximate scattering by lower orders, such as single scattering [15], limiting their validity to weakly scattering environments. Multiple scattering theories [22] are limited to energy transfer equations that describe the propagation as a diffuse wave phenomenon. Numerical methods conversely assume no inherent physical approximations and thereby hold the potential to capture the entire range of physics, including single and multiple scattering. Moreover, analytical methods often rely on effective medium assumptions, whereas numerical methods can provide time-domain data as would be obtained from experimental measurements, which, among other advantages, is a promising feature for wave inversion problems [23].

Various numerical computation schemes exist today, most common in elastodynamics are the finite-difference (FD) [24,25] and the finite-element (FE) [26] methods; others include the spectral method, boundary-element method and the finite-volume method [27]. In addition, the spectral-element method [28,29] is a relatively recent addition to the seismology modelling community which offers certain computational advantages compared with more established techniques. Also worth mentioning are lattice Boltzmann approaches [30], which similarly offer an alternative and may hold promise for future studies of wave scattering.

Focusing on the more established methods, FD and FE techniques have been applied to investigate elastic wave scattering within heterogeneous media, FD being developed first for seismological interests. Early progress includes the study of scattering from Earth-like crustal structures, in the form of one-dimensional-layered media [31–34] and two-dimensional random media [35–39]. A general review is given in [40].

More recently, two-dimensional FE methods have been developed by the NDE community [41,42] with continuing interest [43–46] in the context of ultrasonic waves propagating within polycrystalline materials. These models consider the random spatial fluctuations of local anisotropic elasticity arising from contrasting crystallographic orientations between neighbouring crystallites, also referred to as grains, which when randomly distributed within a polycrystalline aggregate constitute a macroscopically isotropic but scattering medium.

Full three-dimensional models have remained scarce in both seismology and NDE owing to the drastic increase of computational costs compared with two-dimensional ones. Numerical studies of scattering employing three-dimensional FD acoustic codes [47,48] first emerged followed by fully elastic three-dimensional FD [49,50] and three-dimensional FE [51] simulations. Largely enabled by growth in the availability of computational resource, full three-dimensional simulations represent an important milestone as two-dimensional and one-dimensional models are inherently limited in their ability to represent three-dimensional scattering mechanisms encountered in nature [52].

These recent developments now enable, for the first time, full-physics studies of elastic wave scattering. This article aims to establish the validity of the FE method for this purpose through studying its ability to capture fundamental scattering behaviour, here investigated in terms of the attenuation and dispersion of coherent (or ballistic) waves within cubic anisotropic random media, e.g. polycrystals. Whereas recent progress [51] has shown the promise of this technique, here, another order of magnitude increase in the level of computational complexity is achieved to enable the study of remaining and more demanding scattering regimes, and, through consideration of a more statistically significant sample of random events, an unprecedented quantitatively significant accuracy. In addition, the numerically observed scattering is evaluated by a completely independent analytical SOM theory which is the first to be modified to closely match the distribution of length scales, represented by the grain sizes, within the numerical model. Thereby this study is proposed as the first comprehensive and detailed test of second-order scattering theory in polycrystalline materials.

Section 2 describes our computational model from a general perspective in an attempt to also provide an instructive review before it is further developed in §3 for the present interest of obtaining accurate plane wave solutions for a longitudinal bulk wave. In §4, the methodology is employed to numerically evaluate fundamental wave propagation and attenuation behaviour against that expected from established analytical theory.

## 2. Computational model

### (a) Background theory

The basis for a three-dimensional FE formulation to calculate the elastodynamic time response of random heterogeneous media is summarized here. To distinguish from classical deterministic FE, this type of modelling can also be referred to as stochastic FEs as reviewed in [53]. The established [1,2] general numerical formulation, here in the absence of damping, relies on a spatial discretization to compile the global mass and stiffness matrices, *M* and *K*, respectively, to solve the equation of dynamic equilibrium

2.1 $$[M]\ddot{u}+[K]u=F.$$

The stiffness matrix, *K*, represents the stiffness relationship between all points (nodes) of the FE mesh, and it incorporates the material stiffness tensor which represents the material within each element. The material stiffness tensor may be defined to represent any anisotropic material and may vary arbitrarily from element to element; it is thus defined piecewise with step changes on a very small spatial scale. The mass matrix, *M*, similarly expressed as values at the nodes, represents the mass of the elements. The response to an externally applied force, *F*, is represented by displacements *u* and their time derivatives of velocity and acceleration at the nodes. Equation (2.1) is solved using a centralized FD scheme which marches at the time increment, Δ*t*, to explicitly approximate the derivatives of displacement, *u*, at the current, previous and future time step, as denoted, respectively, by *u_t_*, $\;u_{t-\Delta t}$ and $\;u_{t+\Delta t}$ in equation (2.2),

2.2 $${\ddot{u}}_{t}=\frac{1}{\Delta t^{2}}(u_{t-\Delta t}+u_{t+\Delta t}-2u_{t}).$$

Substitution of this into equation (2.1) and rearranging enables the displacement for a future time step to be causally calculated

2.3 $$u_{t+\Delta t}={\left(\frac{1}{\Delta t^{2}}[M]\right)}^{-1}\left(F_{t}-\left(\left[K]-\frac{2}{\Delta t^{2}}[M\right]\right)u_{t}-\left(\frac{1}{\Delta t^{2}}[M]\right)u_{t-\Delta t}\right).$$

This uses a lumped mass approach at the nodes producing a diagonal mass matrix, *M*, to allow for trivial inversion. The advantage of this scheme is that all calculations are local; there is no need to assemble or invert any full system matrix. Further implementation of these equations has been extensively documented [1] and is hence not repeated here. The subsequent sections discuss the lesser-known theory required to accurately model elastic waves within heterogeneous media, particularly for polycrystalline materials [41,42,51].

### (b) Random medium generation

Before spatially discretizing the problem, a numerical method is required to generate our random medium. Many approaches exist to produce either continuous or discretely random media, and each can be mathematically described by their constitutive autocorrelation function, for example Gaussian, exponential or Von Kármán [36]. Here we consider discretely random media, exemplified by multi-phase or porous media and the present case of interest: polycrystalline materials. Voronoi tessellation [54,55] generates numerical models representative of naturally occurring polycrystalline morphologies; this technique is widely established for modelling polycrystalline materials in other research domains including material science [56–58].

**Figure 1.**
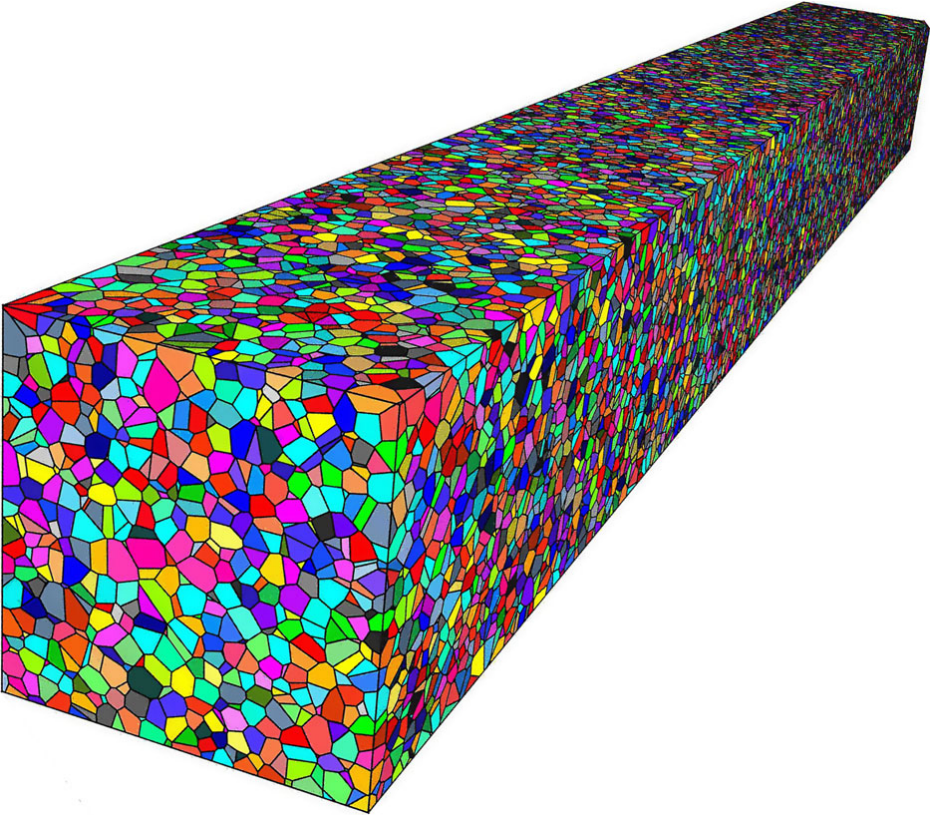
Typical three-dimensional random polycrystalline material tessellated by the Voronoi algorithm and representing over 10^5^ grains. (Online version in colour.)

**Figure 2.**
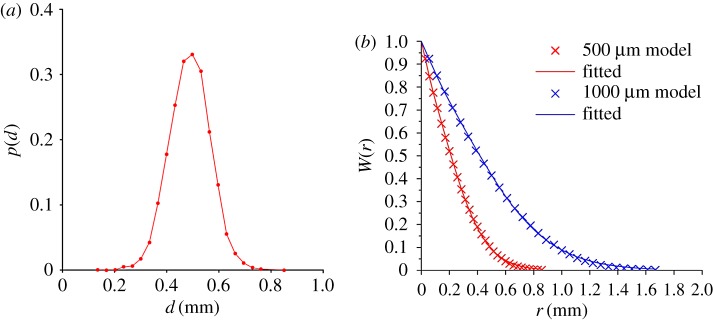
Statistics of two random materials as measured by (*a*) the probability density, *p*(*d*), of the grain size distribution as defined by the cubic root of volume and (*b*) the two-point correlation function, *W*(*r*). (Online version in colour.)

A detailed procedure to generate a Voronoi tessellation can be found in [59]. Following random distribution of points or seeds by a Poisson point process, the Voronoi algorithm produces an outcome as shown in figure 1, representing our three-dimensional polycrystalline material containing over 10^5^ equiaxed grains. The resulting statistical properties of the random medium, which determine the scattering behaviour, depend on the seed randomization procedure. Typical statistics obtained here are specified in figure 2*a* by the grain size distribution, defined as the cubic root of the grain volumes and is further complemented in figure 2*b* by the autocorrelation function, measured by the two-point correlation function (TPC) [60]. As previously mentioned, the autocorrelation function is of particular interest as it is a convenient formulation to describe the properties of a random medium, and is therefore also an essential ingredient for comparison with analytical models which rely on it. Unlike experimental studies where obtaining the TPC from samples presents a cumbersome task, this can be precisely implemented numerically by virtually dropping two random points within the cuboid volume, and through repetition, calculating their probability of existing within a single grain when they are separated by distance *r*—as per the definition of the TPC. Figure 2*b* shows examples of such ‘measured’ functions and the corresponding analytical fit which has been obtained for two three-dimensional models used in subsequent sections.

### (c) Finite-element spatial discretization

Whereas the discretization criteria for conventional elastodynamic modelling are well established avoiding computational errors such as numerical dispersion, additional requirements arise for randomly heterogeneous media. The first involves the added complexity of modelling two length scales, one corresponding to the wave and another to the heterogeneity. A typical wavelength sampling rate at the coarse end of the scale, compromising between solution accuracy and efficiency, consists of 10 grid points per wavelength [26,61]. However, heterogeneities introduce an additional sampling criterion, pertaining to the length scale, *a*. A previous mesh convergence study [51] found that satisfactory convergence to within 1% is achieved with 10 spatial samples per *a* when choosing first-order linear elements [1]*.* When modelling *ka* < 1, this additional sampling criterion significantly increases the computational cost in comparison with conventional wave propagation simulations.

**Figure 3.**
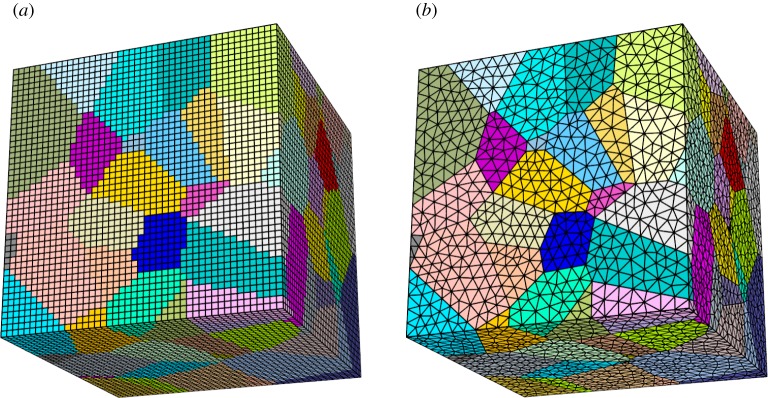
Sample three-dimensional polycrystalline model meshed using (*a*) structured and (*b*) unstructured discretization schemes. (Online version in colour.)

The second concern governs the spatial discretization scheme, whether to adopt a structured or an unstructured mesh (figure 3). Structured grids approximate complex geometries, introducing ‘stair-casing’ effects at oblique boundaries, and thereby require fine sampling in order to achieve a satisfactory spatial representation. Nonetheless, when homogeneously sampled, regions of the model where the geometry of the heterogeneity takes a coarser form may be oversampled, thereby unnecessarily increasing computational cost. Unstructured meshes avoid this by conforming to geometries. However, even these meshes require some regularization to remove finer details from the heterogeneities which would otherwise produce an element size distribution that varies by several orders of magnitude. This is undesirable as it leads to temporal oversampling (see §2d).

In summary, either type of mesh has been shown to perform well under a sufficiently fine discretization [51]; structured meshes are adopted here in conjunction with lowest order, linear elements (i.e. eight-node bricks in three dimensions) for their relative simplicity.

### (d) Polycrystalline material model

Once the problem is discretized, the global *M* and *K* matrices are spatially allocated as dictated by the previously obtained Voronoi tessellation. Each Voronoi cell represents a crystallite whose material properties are obtained by uniformly randomizing (e.g. white noise) every orientation for anisotropy, to produce a heterogeneous stiffness matrix, and, by assigning a uniform material density, a homogeneous mass matrix. Before further detailing the calculation, this present implementation automatically assumes that the grains are perfectly bonded at their boundaries without applying further constraints; this is achieved naturally by the FE representation, because the boundary condition at every boundary between adjacent elements is defined by compatibility (shared displacements) and equilibrium (balance of forces) at the nodes where the elements are joined.

The orientation within a single crystallite is conventionally described by the three Euler angles, and their statistical distribution for an aggregate of crystallites, constituting the polycrystalline sample, is denoted by the orientation distribution function (ODF) [62]. Uniformly distributed ODFs are used in this contribution where every possible crystallographic orientation occurs with an equal probability. This requires the Euler angles to be randomized such that the unit vectors of the final orientations lie equally distributed on the surface of a sphere. This differs from uniformly distributing each Euler angle, which would yield a higher probability at the poles of the ODF [42]. The correct implementation of a macroscopically isotropic material can be verified numerically by simulating and confirming that the wave velocity through the model remains unchanged in the three principal *x-*, *y-*, *z*-directions. Conversely, preferential ODFs, referred to as textured materials, have also been implemented into FE codes by Chassignole *et al*. [63] to study ultrasonic wave propagation within welds.

Although the method can accommodate any crystal symmetry, all example cases modelled here consist of cubic anisotropic materials; the general crystal stiffness tensor in its simplest principal axis formulation is given in equation (2.4)

2.4 $$C_{\mathrm{cubic}}=\left(\begin{array}{cccccc} C_{11} & C_{12} & C_{12} & 0 & 0 & 0\\ C_{12} & C_{11} & C_{12} & 0 & 0 & 0\\ C_{12} & C_{12} & C_{11} & 0 & 0 & 0\\ 0 & 0 & 0 & C_{44} & 0 & 0\\ 0 & 0 & 0 & 0 & C_{44} & 0\\ 0 & 0 & 0 & 0 & 0 & C_{44} \end{array}\right),$$

where *C*_cubic_ holds all elastic properties to describe the element stiffness matrix as a subset of the global *K* matrix as described in [1]. Inconel and aluminium are numerically implemented as examples in this study to represent a strongly and a weakly scattering material, respectively, as dictated by the [64] anisotropy coefficient, $A_{\mathrm{cubic}}=2C_{44}/C_{11}-C_{12}$. Their material properties are detailed in table 1. In addition, a more general representation is given by the universal anisotropy [65], *A*_u_, to enable comparison with other crystal symmetries.

**Table 1.** Material properties used to represent Inconel and aluminium.

material	*A*	*A*_u_	*ρ* (kg m^−3^)	*C*_11_ (GPa)	*C*_12_ (GPa)	*C*_44_ (GPa)
Inconel	2.8	1.4	8000	234.6	145.4	126.2
aluminium	1.2	0.04	2700	103.4	57.1	28.6

### (e) Loading conditions

Loading conditions serve to excite the desired wave and usually involve modelling spatially finite sources by imposing either a prescribed displacement boundary condition or a force loading constraint. The latter is employed here but in the particular interest to achieve a bulk wave representation within an infinite volume: namely the plane wave case.

**Figure 4.**
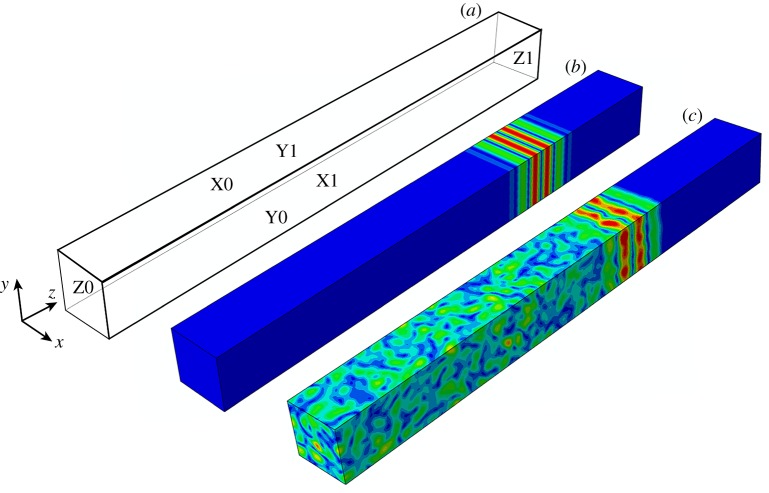
(*a*) FE cuboid model layout labelling the exposed Z0, Y1, X1 and hidden Z1, Y0, X0 surfaces; (*b*) plane wave propagation within a monocrystalline anisotropic solid; (*c*) plane wave propagation and scattering within a polycrystalline solid. In both cases, the plane wave is propagating in the *z*-direction. (Online version in colour.)

The chosen set-up for the studies reported here considers, without loss of generality, a longitudinal plane wave propagating in the *z*-direction within a cuboid with outer surfaces X0, X1, Y0, Y1, Z0 and Z1, as depicted in figure 4. Generating a plane wave in this scenario requires exciting all the nodes which lie on an external surface, in this case the Z0 surface, with a *z*-direction force profiled by a three-cycle tone burst, thereby generating a longitudinal wave. Whether these loading conditions achieve plane wave representation depends largely on the implementation of the boundary conditions.

### (f) Boundary conditions

In our case, boundary conditions serve to accommodate the desired plane wave mode; we will make use of two alternative kinds, each applied at the exterior surfaces of the domain: symmetry boundary conditions (SBCs) and periodic boundary conditions (PBCs), where the former is more commonplace [51] in wave propagation simulations. Later, in §3, we investigate the use of each of these kinds of boundary conditions. Here we just describe how they are defined.

**Figure 5.**
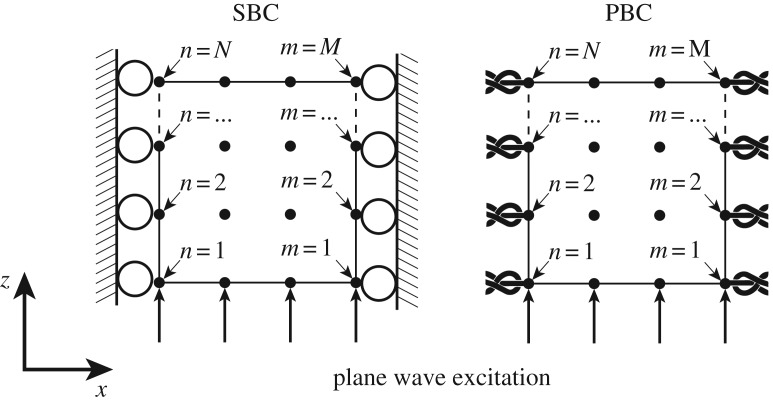
Two-dimensional schematic of periodic (PBC) and symmetry (SBC) boundary conditions applied to the nodes (represented by dots) on the external edges of the model to accommodate a plane wave propagating in the *z*-direction.

SBCs define nodal displacements (and thus also velocities and accelerations) to be zero in the direction normal to the four outer surfaces that are lateral to the wave propagation direction, namely faces X0, X1, Y0 and Y1 in figure 4. For a single surface, e.g. X0, this entails setting $u_{x}^{n}=0$ for all *N* nodes, where $u_{x}^{n}$ denotes displacement at the *n*th node in the *x*-direction. This is illustrated, for clarity, in the two-dimensional case in figure 5 which collapses the surface X0 and X1 to a line with *N* and *M* nodes, respectively.

Such boundary conditions are strictly correct to accommodate a plane wave within an isotropic homogeneous medium; however, this is not always the case for anisotropic media. To illustrate this, consider a homogeneous anisotropic medium (e.g. monocrystal). In this simple case, the plane wave representation breaks down whenever the particle displacement is not parallel to the direction of wave propagation, such that a skew angle exists between the group and phase velocity vectors (a diagrammatic explanation can be found in [66]). At such orientations, the longitudinal wave manifests as a quasi-longitudinal wave, and this is the case for the vast majority (all but 26 for cubic) of the propagation directions within an anisotropic medium. In these cases, the wave motion is not mappable to a single Cartesian coordinate (at least not in a cuboid), and, therefore, no displacement constraint can be assigned which constrains displacement perpendicular to the direction of motion to accommodate a plane wave.

This same situation arises along the boundary of a polycrystalline material, thereby introducing errors. However, it remains unknown whether this has a significant impact on the result, as an averaging effect takes place owing to the random orientations existing along the boundary. This is subject to investigation in §3b.

Contrarily, PBCs are capable of accommodating quasi-longitudinal plane waves. PBCs tie the displacements (and thus velocities and accelerations) of the *n*th node which lies on one extremity of the model (on, for example, surface X0) to its respective partner *m*th node, located in the same location on the opposite extremity (e.g. surface X1). This condition is repeated to tie surface pairs, (Y0,Y1) and (X0,X1) together. The implementation fixes the displacement of partnering nodes to be equal for all degrees of freedom (d.f.), e.g. $u_{x,y,z}^{n}-u_{x,y,z}^{m}=0$, as illustrated in figure 5 for the two-dimensional case.

PBCs achieve an infinite representation by repeating the finite model, and thereby obtain a plane wave solution, regardless of the direction of wave motion. However, the PBC approach also holds a limitation as it represents a material whose grains have a repeating periodic shape and orientation; thus, it works perfectly for a monocrystalline material, but only approximately for a polycrystal. Nevertheless, we can interpret it as a more general boundary condition than the SBC, because the displacements are controlled but not necessarily zero, and thus there is reason to expect that it might give an improved performance.

### (g) Time marching

**Figure 6.**
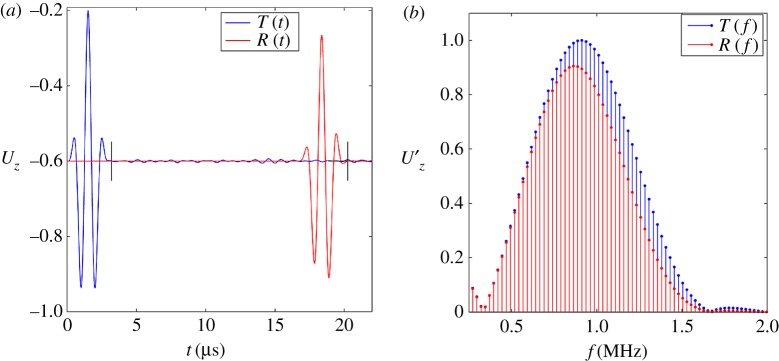
Typical *z*-displacement amplitude, *U_z_*, for the excitation, *T*, and received, *R*, signals plotted in the (*a*) time domain, *t,* and (*b*) frequency domain, *f*. The typical time window (starting at *t* = 0) used for *T* and *R* are indicated in (*a*) by vertical black lines. (Online version in colour.)

Upon completion of the above steps, the equation, as shown in (2.1), is configured and can be incrementally solved for displacement. Although the solution requires solving for all nodal displacements, usually only a portion of them are monitored, which can be chosen to occur at any location within the model. In this case, it is decided to monitor the displacements of all nodes which lie on the excitation, Z0, and receiving, Z1, surfaces. A typical displacement time trace is shown in figure 6*a* and is available through the electronic supplementary material.

The explicit solving scheme requires defining the time step, Δ*t,* by the well-known Courant–Friedrichs–Levy [67] stability condition, shown in equation (2.5), where $\Delta l_{-}$ is the smallest spatial dimension between two nodes and *V_+_* is the maximum wave velocity,

2.5 $$\Delta t<\frac{\Delta l_{-}}{V_{+}}.$$

When modelling heterogeneous materials, this latter parameter *V_+_* must be defined carefully owing to the velocity fluctuations throughout the model. For an anisotropic material, the maximum wave velocity can be found from the phase velocity surface, more commonly presented by its reciprocal, the slowness surface [66].

Although the requirement for stability must be met, it is not advisable to exceed it, i.e. to use much smaller steps, as oversampling increases both amplitude errors [61] and computational cost. This is often unavoidable when using unstructured meshes where the distribution of element sizes spans several orders of magnitude, as discussed in §2b.

Typical solving times of approximately 100 min are achieved here for the models outlined in table 2. Given the vast (300 million+) number of d.f. (per time step), and by 2016 standards, the solution time is significantly accelerated by the use of 8× Tesla K80 GPUs and the Pogo software [68].

**Table 2.** Model details used for three-dimensional studies.

model label	3D-N5120	3D-N115200	3D-N14400	3D-N11520
centre frequency	2 MHz	1–3 MHz	1–15 MHz	3–15 MHz
dimensions (*w *× *h *× *l*)	4 mm × 4 mm × 40 mm	12 mm × 12 mm × 100 mm	12 mm × 12 mm × 100 mm	12 mm × 12 mm × 10 mm
no. of grains	5120	115 200	14 400	11 520
grain size	500 µm	500 µm	1000 µm	500 µm
d.f.	16 × 10^6^	345 × 10^6^	345 × 10^6^	278 × 10^6^
material	Inconel	Inconel	aluminium	Inconel
boundary condition	PBC/SBC	SBC	SBC	SBC

## 3. Methodology development and validation

In this section, we extend the aforementioned methodologies to achieve accurate simulations of plane waves within heterogeneous media. The plane wave case, in particular, is valuable to study scattering phenomena as, being void of diffraction effects associated with waves emerging from spatially finite sources such as real transducer set-ups, it enables direct comparison with established analytical theories.

### (a) General model and calculations

The general model involves propagating plane waves within a rectangle in two dimensions or a cuboid in two dimensions of polycrystalline material, as detailed in §2, illustrated in figure 4, and further detailed in tables 2 and 3. The excited waves comprise a three-cycle tone burst (figure 5*a*) at various centre frequencies in order to investigate different scattering regimes. The time-domain *z*-displacement of the transmitted signal, *T*(*t*), and received signal, *R*(*t*), are obtained from the mean nodal displacement at each step in time across surface Z0 and Z1, respectively (shown in figure 6).

**Table 3.** Model details used for two-dimensional parametric studies.

model label	2D-N6000
centre frequency	2 MHz
dimensions (*l *× *w*)	50 mm × 30 mm
no. of grains	6000
grain size	500 (µm)
material	Inconel

The output time-domain signals *T*(*t*) and *R*(*t*) (exemplified in figure 6*a*) are further processed to calculate the attenuation and phase velocity, each as a function of frequency over the bandwidth of the signal. The method [69] involves obtaining the ratio of spectral amplitudes and the difference in unwrapped phase through fast Fourier transforming each signal. Figure 6*b* displays a typical frequency amplitude spectrum of *T*(*f*) and of *R*(*f*). The loss in amplitude incurred during the propagation length can be seen in both parts of the figure; it can be seen to be particularly strong at high frequency.

### (b) Plane waves in anisotropic media

#### (i) Monocrystalline medium

**Figure 7.**
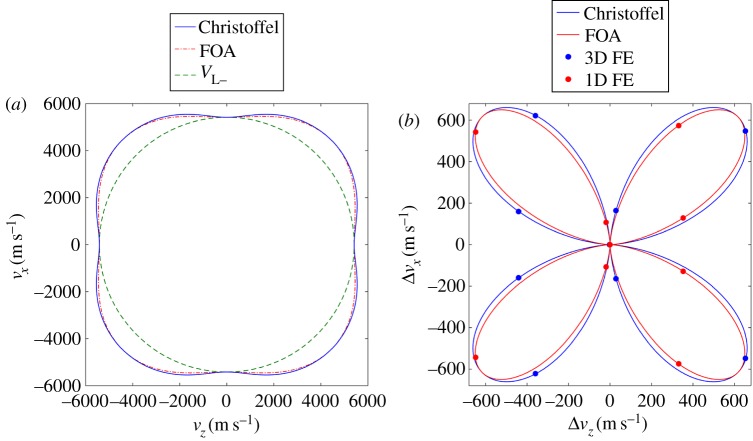
(*a*) Phase velocity surface of a longitudinal wave in a cubic anisotropic medium comparing theoretical solutions of the three-dimensional Christoffel equation and a one-dimensional first-order approximation (FOA). The minimum velocity, *V*_L_, illustrates the isotropic case. Both are replotted in (*b)* as a relative change away from *V*_L*−*_ including the FE results in three dimensions (using PBC) and one dimension. Average error between the analytical and the numerically calculated phase velocity is 0.02%. (Online version in colour.)

Before considering the full complexity of a polycrystalline medium, we validate our numerical methodology by evaluation against the analytically obtained phase velocity for a plane wave propagating at various orientations within a monocrystalline solid, depicted by the phase velocity surface shown in figure 7*a*. Two representations are considered: (i) a simplified one-dimensional case and (ii) the full three-dimensional case as given by the Christoffel equation [66]; both are evaluated numerically and analytically.

The numerical procedure for either case evaluates the phase velocity following rotation of the crystal orientation through the second Euler angle (*y*-axis shown in figure 4), in 40° intervals until the full 360° range is considered (the direction of this rotation is reversed between the two cases to consider separate points on the wave velocity surface). The full details of the numerical model, labelled 3D-N5120, can be found in table 2 (in this case ignoring ‘no. of grains’ and ‘grain size’).

*One-dimensional solution:* first, we consider the simplest case, the one-dimensional solution that confines motion to occur only in the *z*-direction, realized by constraining all nodal displacements in the *x-* and *y*-dimensions throughout the volume of the cuboid. As this eliminates all but the first of the stiffness coefficients (equation (2.2)), the numerical solution can be analytically verified by a first-order approximation (FOA) $V_{L}=\sqrt{{C^{\prime }}_{11}/\rho },$ where $C_{11}^{\prime }$ represents the rotated *C*_11_ component of the stiffness matrix and *ρ* the material density—its solution is plotted in figure 7*a*.

The numerical and analytical results are plotted in figure 7*b* as the difference from the minimum phase velocity (shown in figure 7*a*). The graph shows excellent agreement between the FE and the FOA solution. Before considering the three-dimensional solution, we can therefore note that this simple one-dimensional case verifies our numerical implementation of the crystallographic orientation rotation calculations and the post-processing methodology to extract the frequency-dependent phase velocity.

*Three-dimensional solution*: the second representation involves the full three-dimensional solution for wave propagation within an anisotropic solid, as given by the well-established Christoffel equation (eq. 7.19 in [66]). Its solution provides the wave velocity characteristics in a given direction for the three propagation modes: one quasi-longitudinal and two quasi-transverse waves. The displacement vector in the quasi-longitudinal wave is at a non-zero angle (skew angle) to the direction of wave propagation. Along certain directions, those quasi-waves become pure longitudinal and transverse modes with the displacement vectors in the usual polarization direction as in an isotropic material. It is important to note that a single mode excitation (in this case, the quasi-longitudinal mode) requires the excitation vector to match with the eigenvector obtained from the solution to the Christoffel equation [66]. When rotating the crystal about the *y*-axis, and when a skew angle exists, the required excitation is thus composed of both *z* and *x* components. The resulting single quasi-longitudinal mode travelling along the cuboid is confirmed by figure 4*b* for the example of a plane wave propagating at a 22° orientation, producing a skew angle of 11.8°. The colour contours show the phase front to be in the *xy* plane, with clean propagation in the *z*-direction. The particle motion of this wave is in both the *z-* and *x*-directions, therefore requiring PBC on the XO, X1, Y0, Y1 surfaces as described in §2b.

As in §3b, figure 7 plots the numerically and analytically obtained phase velocity surface for the three-dimensional case. First, comparing the one- and three-dimensional analytical solutions, the FOA and the Christoffel equation can be seen to deviate at propagation directions where a skew angle manifests and hence, at least for a cubic material, the FOA result matches the full three-dimensional result when the skew angle is zero. In addition, it is interesting to observe that the constrained solution (one-dimensional case) produces slower wave speeds than the full three-dimensional case, which seems non-intuitive when considering that displacement constraints are usually associated with the introduction of stiffness into a model.

The average error between the three-dimensional numerical and analytical results, in the region of 0.02%, shows that this simulation is extremely accurate in comparison with the expected accuracy of around 0.5% reported in [70]. This completes our validation of generating a plane wave within a monocrystalline anisotropic material—next we consider the polycrystalline case.

#### (ii) Polycrystalline medium

The boundary conditions required to obtain a plane wave solution within a polycrystalline material are hereby investigated. In addition to the previously investigated PBC, SBCs are considered for their relative simplicity. As mentioned previously, SBCs introduce an error for displacements along the boundary as they constrain displacements which would be free to occur in the PBC case. However, it is postulated that this error will average out when considering a sufficiently wide ensemble of randomly oriented grains. The full details of the numerical model, labelled ‘3D-N5120’, can be found in table 2.

**Figure 8.**
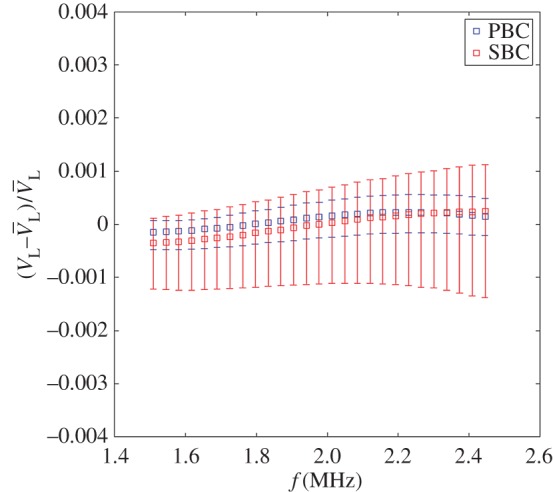
Effects of periodic and symmetry boundary conditions on the obtained phase velocity dispersion of longitudinal waves within a polycrystalline medium. Squares represent the mean value and bars represent the minimum and maximum found from three independent realizations. Velocities are normalized against ${\bar{V}}_{L}$, representing the mean velocity from this data. (Online version in colour.)

Figure 8 plots the phase velocity for a polycrystalline material with three realizations of random orientations (see §3c(ii)), repeated for both SBC and PBC exterior boundaries. Unlike the monocrystalline case, no significant difference can be found between the results for the two kinds of boundary conditions, although the data spread slightly wider in the SBC case. These results suffice to validate the use of either PBCs or SBCs for models of this size in future studies.

### (c) Multiple realizations of randomly orientated polycrystals

Owing to the random nature of scattering, statistical considerations are often desirable. This can be achieved relatively easily numerically by considering the scattering response from multiple randomly generated but statistically identical materials (as outlined in §2d). However, this can significantly increase the required computation as a considerable number of independent meshed models and measurements may be needed before satisfactory confidence bounds are found. Instead, a more efficient methodology is developed here in an attempt to enable a higher statistical significance and accuracy for the results in §4.

#### (i) Two-dimensional validation

A relatively simple test is performed to verify whether savings can be derived from solely reshuffling the random grain orientations within an existing model (existing set of grains) in order to obtain an independent measurement, or whether it is necessary to randomize both grain orientations and grain distributions which would require newly meshing a model for each realization. Two metrics are used for the analysis, both are calculated from the Hilbert envelope of the *T*(*t*) and *R*(*t*) signals. Namely, the time-domain wave velocity, *V*, and time-domain amplitude, *A,* are obtained by observing the difference in time and amplitude, respectively, between the two Hilbert peaks of *T*(*t*) and *R*(*t*). The distribution of amplitude and wave velocity is subsequently calculated from approximately 100 randomly generated materials using both schemes. Details are provided in table 3. Owing to this large number of simulations and the relatively high computational cost of three-dimensional models, the models are limited to two dimensions by collapsing the *y*-dimension by plane strain assumptions (*u_y_* = 0). The full details of the numerical model can be found in table 3.

**Figure 9.**
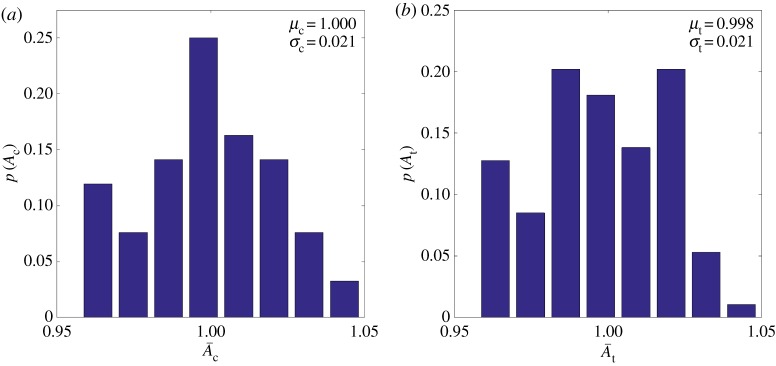
Distribution of amplitude when randomizing (*a*) both orientation and morphology (representing the control case, *c*) and (*b*) solely orientations representing the test case *t* where *µ* denotes the mean and *σ* the standard deviation. In each case, normalization is performed using $\bar{A_{c}}$. (Online version in colour.)

The resulting amplitude statistics for each approach are shown in figure 9. For the amplitude results, the agreement of the mean, *µ*, is to within 0.2% and the standard deviation, *σ*, is identical to within three decimal places. Both velocity metrics are within 0.01% agreement albeit showing little standard deviation, 0.07% and 0.08% away from the mean and hence are not plotted here. Together, this establishes that the statistics that are reproduced by either scheme, i.e. the mean and standard deviation of each distribution, are almost identical. Hence, we are able to conclude that independently simulated cases can be created by simply re-randomizing the crystal orientations while retaining the same grain geometry. This presents a significant saving in computational cost as the meshing calculations need not be repeated.

#### (ii) Three-dimensional results

The previous results are tested in three dimensions to provide confidence bounds on the measurement of interest: attenuation and phase velocity; both are calculated for 20 random realizations of grains within the 3D-N115200 model to consider their standard deviation.

**Figure 10.**
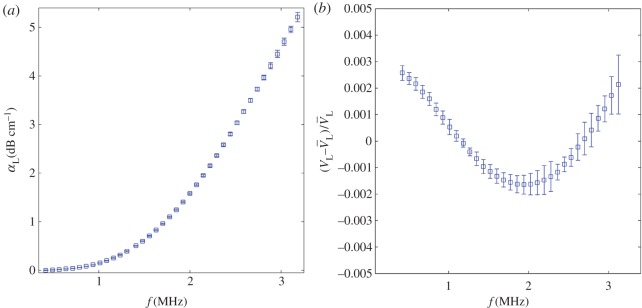
Standard deviation (bar) and mean (square) plotted for (*a*) attenuation and (*b*) phase velocity (normalized against the mean velocity, ${\bar{V}}_{L}$) for 20 random realizations of a polycrystalline material. (Online version in colour.)

The results are shown in figure 10, where, firstly, the standard deviation bars of attenuation can be observed to be insignificant in comparison with the scale of growth within the frequency range considered. The phase velocity remains largely unchanged however, and, thereby, the standard deviations bars seem more substantial. Even so, both metrics show standard deviation which is deemed largely satisfactory for the investigation in §4. We briefly discuss the considerations which govern the measurement statistics.

When aiming to evaluate the characteristics of an infinite plane wave using a finite size polycrystal model, an approximation is introduced as the measured spatially coherent finite wave will contain a contribution from undesired incoherent waves—analogous to backscattering noise encountered in physical measurements. This form of noise corrupts the amplitude and phase of the sampled signal, an effect that becomes more prominent as frequency increases. Thus, the first requirement for accurate calculations entails maximizing the potential for spatial averaging; this can be achieved by either increasing the lateral extent of the model or (as discussed previously) by considering multiple independent realizations and either ensemble averaging the coherent waves or averaging the resulting attenuation and dispersion from each realization. Both schemes have been tested and were found to reveal almost identical results; the latter provides the additional information of the measure of the variance, so it is adopted here.

In addition, the numerical calculations have requirements analogous to those of experimental measurements of attenuation. Namely, errors arising from experimental measurement are minimized when the total attenuation is around one Neper [69], and deviation from this value leads to a magnification of the error during the attenuation calculations. Extreme values of attenuation on either end of the scale therefore result in larger computational errors; for the numerical models considered here good results were found when maintaining amplitude losses between 6 and 40 dB. The level of attenuation can be controlled by adjusting the propagation length or the grain size.

The abovementioned considerations led to the development of separate models for the following investigation, 3D-N115200, 3D-N11520 and 3D-N14400 shown in table 2, where, for instance, the grain size is increased in the latter to enable the study of attenuation effects in weakly scattering materials.

## 4. Results: attenuation and phase velocity

The FE results are evaluated by comparison of the observed scattering-induced attenuation and velocity dispersion behaviour with that predicted by well-established analytical theory—in this case, a SOM. A weakly and a strongly scattering polycrystalline material is considered with properties (table 1) representative of aluminium and Inconel, respectively.

The analytical calculations involve a second-order approximation, using the underlying assumptions to the well-established Stanke & Kino [8] and Weaver-type [14] models. However, instead of a typical inverse exponential autocorrelation function, in the form exp(−*r*/*a*), the SOM here assumes a modified function in the form of an exponent series (see equation (4.1)) that is an analytical fit (with *j* = 4; shown in figure 2*b*) of the TPC which is numerically and precisely obtained from our randomly generated materials. Implementation of the modified TPC requires transformation to a spectral representation before substitution into the dispersion equation for the perturbed wavenumber detailed in [19],

4.1 $$W(\text{r})=\sum_{i=1}^{j}A_{i}\exp\left(-\frac{r}{a_{i}}\right).$$

**Figure 11.**
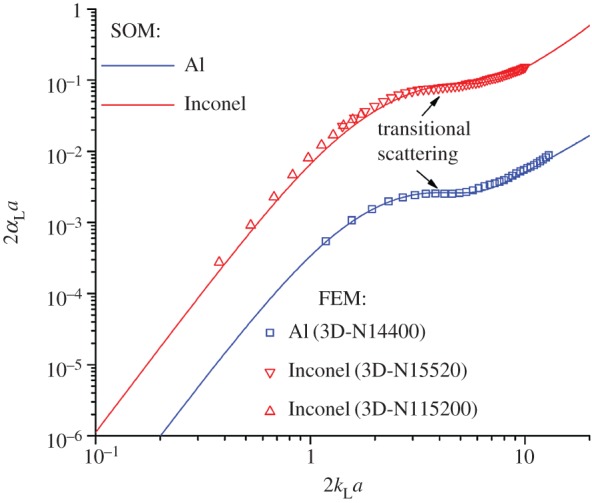
Scattering induced attenuation of longitudinal wave within polycrystalline medium. The normalized domain [8]: attenuation, *αa*, where *α* is attenuation and, *a*, the mean grain size, versus normalized propagation constant, *ka*, where *k* denotes wavenumber. Results are obtained using analytical (SOM) and numerical (FEM) models for polycrystalline aluminium and Inconel. (Online version in colour.)

**Figure 12.**
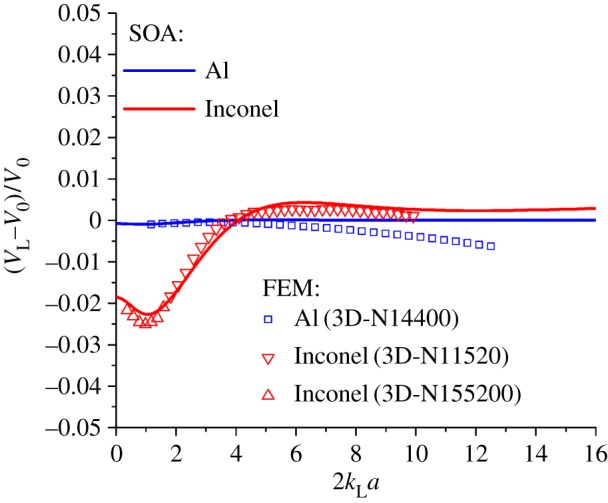
Phase velocity of a longitudinal wave within a polycrystalline medium. The normalized domain [8]: phase velocity, *V*_L_, and the reference Voigt velocity, *V*_0_, versus normalized propagation constant, *ka*, where *k* denotes wavenumber and *a* denotes the mean grain size. Results are obtained using analytical (SOM) and numerical (FEM) models for polycrystalline aluminium and Inconel. (Online version in colour.)

The numerical calculations rely on the previously outlined methodology to achieve an equivalent and accurate plane wave solution. Complete details of the three-dimensional models, labelled ‘3D-N14400’, ‘3D-N11520’ and ‘3D-N115200’, can be found in table 2 along with the material properties provided in table 1. The models differ slightly in parameters such as grain size and mesh refinement to enable investigation of a large wavelength spectrum of scattering while maintaining the absolute attenuation within desirable bounds for both materials as discussed in §2 g. In addition, the results data in the subsequent figures 11 and 12 are labelled so as to indicate which particular model is employed.

By these measures, the equivalency of the numerical and analytical models on which the comparison is based is significantly improved, far beyond what is practically possible, using experimental techniques or beyond what was previously possible in prior numerical studies. The evaluation is thus expected to represent the best possible wave propagation and scattering comparison. The only remaining contrast is the assumption of a second-order approximation to scattering within the analytical model, whereas the numerical model embodies full multiple scattering. The consideration of alternative scattering theories such as [18,19] and the validity of their different approximations is beyond the current scope.

### (a) Weak scattering case: aluminium

We first investigate a relatively weakly scattering material, aluminium. Given this is the lower anisotropy case, it is expected to reveal the smallest discrepancies between the full-physics simulations and analytical approximations which are limited to second-order material perturbations and therefore also in terms of the account for multiple scattering. Even so, as discussed in §3c(ii), the FE models are not infinite in width and hence contain some noise.

The normalized attenuation coefficient for longitudinal waves in polycrystalline aluminium is plotted in figure 11, as calculated by both models. The Rayleigh and stochastic scattering can be seen to be well represented by the numerical model, with an average agreement of the order of 2% (peak approx. 10%) for the particular range tested here. This is considered to be well within satisfactory bounds; moreover, it is believed to be the first independent reproduction of the transitional scattering regime, which has been particularly challenging to confirm using experimental techniques. The complexity of this regime is the longitudinal wave scattering transition from scattering into predominantly transverse waves at low *ka* (Rayleigh regime) to scattering into predominantly longitudinal waves at larger *ka* (stochastic regime).

The adjoint phase velocity results are plotted in figure 12 after normalization against the Voigt velocity; an average velocity is obtained from the mean elastic tensor for a Voigt reference medium (for an excellent review, see [18]). Trend matching of both methods is again found to be excellent with a discrepancy which begins at practically 0% and slowly grows at an accelerating pace towards a peak of 0.7%. Given the nature of this discrepancy, it is likely to be caused by numerical dispersion as relative discretization worsens as *ka* increases.

### (b) Strong scattering case: Inconel

In comparison with the previous case, Inconel represents a strongly scattering material. The normalized attenuation coefficient for longitudinal waves within polycrystalline Inconel is shown in figure 11. The agreement for attenuation between the numerical and analytical results remains convincing, with the transitional behaviour being well reproduced and an average relative difference of the order of 10%. This disagreement remains within acceptable bounds. In comparison with the lower anisotropy, aluminium, case, the larger discrepancy here is to be expected owing to the higher levels of noise encountered by the increased scattering activity, and also owing to a larger distribution of wavelengths, which ultimately causes the mesh to be decreasingly well sampled. Lastly, the overlap between two Inconel models which produce almost identical attenuation values provides further confidence in the results.

The phase velocity results in figure 12 equally show excellent agreement; the average difference is in the region of 0.2% and fluctuates as both a positive and negative difference.

## 5. Conclusion

This article sets out to develop and establish an accurate numerical methodology to study wave propagation and scattering in heterogeneous media. A general FE formulation is outlined which solves the three-dimensional elastodynamic propagation problem within media that present random variations in their elastic properties; the example of cubic anisotropic polycrystalline materials is adopted which employs the Voronoi algorithm to numerically generate representative morphologies. Further development and validation of the method includes achieving an unbounded plane wave solution by implementation of periodic and SBCs and, in the interest of efficient computation, evidence of achieving a truly independent realization of the random medium by solely re-randomizing the elastic fluctuations (i.e. the anisotropic orientations) while maintaining their spatial distribution (i.e. grain geometry), thereby avoiding computationally intensive re-calculations of a mesh.

The numerical methodology is evaluated by comparison of the observed scattering behaviour with that predicted by well-established analytical theory. The analytical model bases itself on the Stanke & Kino and Weaver-type SOMs, but implements a modified autocorrelation function to precisely match the statistics of the particular random medium under consideration.

Scattering behaviour is studied within both a weakly and strongly scattering cubic anisotropic material, across a spectrum of *ka* that varies by an order of magnitude such that the Rayleigh, transitional and stochastic scattering regimes are all visited. For the different cases considered here, the attenuation agreement between theory and numerical results was found to be excellent, with an average difference of the order of 10%. It is believed that this is the first quantitative validation that gives significant support towards the existence of the longitudinal wave attenuation hump related to the transitional scattering regime. Similarly, the dispersive characteristics of the phase velocity were found to be accurately captured, with an agreement of the order of 0.5% in wave speed.

It is thereby believed this article has successfully demonstrated the strength and versatility of FE modelling in studying complex physical behaviours such as the elastic wave propagation and scattering within heterogeneous media.

## Supplementary Material

Time-trace data 1

## Supplementary Material

Time-trace data 2

## Supplementary Material

Time-trace data 3

## Data accessibility

An example of the output simulation data is available in accompaniment of this article. Time-trace data (as referred to *R*(*t*) and *T*(*t*) within the article) are included as MATLAB .mat files for the ‘3D-N115200’ model.
